# Predicted Roles of the Uncharacterized Clustered Genes in Aflatoxin Biosynthesis

**DOI:** 10.3390/toxins1010037

**Published:** 2009-09-25

**Authors:** Kenneth C. Ehrlich

**Affiliations:** Southern Regional Research Center, ARS, USDA/1100 Robert E. Lee Blvd, New Orleans, LA 70124, USA; Email: ken.ehrlich@ars.usda.gov; Tel.: +1-504-286-4369; Fax: +1-504-286-4419

**Keywords:** biosynthesis, oxidation, enzymes, *Aspergillus*, secondary metabolism

## Abstract

Biosynthesis of the toxic and carcinogenic aflatoxins (AFs) requires the activity of more than 27 enzymes. The roles in biosynthesis of newly described enzymes are discussed in this review. We suggest that HypC catalyzes the oxidation of norsolorinic acid anthrone; AvfA (AflI), the ring-closure step in formation of hydroxyversicolorone; HypB, the second oxidation step in conversion of *O*-methylsterigmatocystin to AF; and HypE and NorA (AflE), the final two steps in AFB_1_ formation. HypD, an integral membrane protein, affects fungal development and lowers AF production while AflJ (AflS), has a partial methyltransferase domain that may be important in its function as a transcriptional co-activator.

## 1. Introduction

The aflatoxins (AFs) are the most studied of all of the fungal toxins (for recent reviews, see references [1,2,3,4,5,6,7,8,9,10]). Not only are they powerful toxins to some animals, they are among the most carcinogenic naturally occurring compounds. The species of *Aspergillus* that produce aflatoxins are found on all continents with a temperate climate [11]. They are commonly found in the soil where they survive on many plant and animal debris and insect waste [12]. Under favorable conditions for the fungi, they can also become pathogens of plant seeds. They reproduce by forming spores and sclerotia. These reproductive structures are extremely hardy and can survive in the soil for years [13]. Because of their toxicity, the amounts of AFs allowed in crops intended for human and animal consumption are carefully regulated [14]. The levels allowed range from 20 to 200 ppb in corn, cottonseed and tree and ground nuts in the United States and most other countries [15]. 

The biosynthesis of AFs has been extensively studied [3,16]. The genes that encode enzymes and transcription regulatory factors for their biosynthesis are maintained on a 75 kDa subtelomeric gene cluster on chromosome 3 [17,18]. Approximately 29 genes have been identified in the cluster. The characterized genes encode enzymes involved in biosynthetic steps starting from the precursors, malonyl and acetylCoA. Since their discovery in 1960, the main biosynthetic steps have been identified and detailed reviews of the chemistry and biosynthesis of AF formation are available [4,16,19,20,21,22]. It is the intention of this review to focus only on the proteins whose roles in biosynthesis have not been adequately described in previous reviews. Surprisingly, detailed characterization of many of the enzymes involved in the catalysis of the biosynthetic steps is sorely lacking even when the genes involved in biosynthesis were characterized in detail. In fact, only 11 of the 29 enzymes and regulatory factors involved in the biosynthesis have been isolated and their activity characterized by kinetic studies, and, of these, only some have been assigned to specific catalytic steps in the biosynthesis pathway. Because of this inadequate knowledge of protein function, some of this review’s conclusions on the role of the enzymes will be, of necessity, speculative. However, because the literature regarding AF biosynthesis is so extensive, placing the newly discovered hypothetical enzymes and some of the older, as yet, uncharacterized enzymes into the biosynthetic puzzle should help to better understand the complexity of the biosynthesis and the logic behind some of these steps.

## 2. General Considerations Concerning Aflatoxin Biosynthesis

### 2.1. Gene Clusters and Aflatoxin Biosynthesis

**Figure 1:**
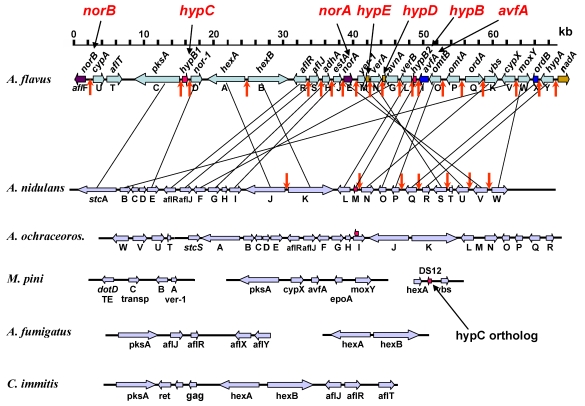
Comparison of secondary metabolite gene clusters in six species of fungi that contain some of the genes related to AF production. The genes whose function is considered in this paper are shown above the *A. flavus* AF cluster schematic. Vertical lines link gene orthologs in the ST and AF clusters. Vertical red arrows are known AflR-binding sites.

It is fortunate for understanding the function of the genes and proteins involved in AF biosynthesis in *A. flavus* and *A. parasiticus* that all of the genes are maintained in a 75 kbp gene cluster [23,24] (Figure 1). The cluster contains not only the genes that encode the enzymes necessary for biosynthesis, but also the genes that encode the two key regulatory proteins necessary for transcription. The AF cluster like so many gene clusters in eukaryotes represents a regulatory island, presumably, to allow coordinated production of the catalytic machinery necessary for all of the steps in biosynthesis [25,26]. Related clusters are also shown in Figure 1 [1,27,28,29,30,31]. Three of these, the *A. nidulans* ST cluster, the *A. ochraceoroseus* AF cluster and the *Mycosphaerella pini* dothistromin biosynthesis clusters encode some of the same biosynthetic proteins as do the genes in the AF cluster. Three separated regions form the dothistromin cluster. Since no transcription regulatory protein was identified in the dothistromin clusters, the regulation of expression of genes in this cluster may be different from that of the AF and ST clusters. The *A. ochraceoroseus* AF cluster possesses most but not all of the AF biosynthesis genes. Genes for conversion of ST to AF formation are encoded by genes outside of the cluster. Two fungi known to be highly virulent to humans, namely, *A. fumigatus* and *Coccidioides posadasii/immitis,* have gene clusters resembling the AF/ST cluster. These small clusters may be capable of producing an anthraquinone precursor, but no metabolites related to these clusters have yet been characterized.

### 2.2. Problems in Gene Characterization

Conversion of precursors to AFs involves several major steps: 1) formation of the anthraquinone polyketide; 2) a series of oxidations of the C-6 hydrocarbon side chain on the anthraquinone to convert the first stable metabolite, norsolorinic acid (NA) to averufin (AVF) and versicolorin A (VERA); 3) oxidation of the anthraquinone moiety and rearrangement of VERA to the xanthone, sterigmatocystin (ST); 4) oxidation and rearrangement of the xanthone to the AF coumarin nucleus. There is substantial evidence that the key enzymes involved in the various conversion steps are coordinately regulated and that, therefore, most of the polyketide “decorating” enzymes are simultaneously available for carrying out the steps in the biosynthesis [20]. This observation implies that the sequence of reductions, oxidations, hydrolyses, and rearrangements is mainly dictated by the differential chemical susceptibilities of the different portions of the initially formed anthraquinone, rather than by its position on a chemical assembly line, where the sequential steps are dictated by relationships of the catalytic proteins to one another, as may be the case for the formation of some of the secondary metabolites in bacteria [32]. That being said, it is possible that the gene arrangement has some role to play in the sequential attack on the precursors as they are formed and that the precursors, while susceptible to other oxidative enzymes in the cell, are shielded from others. The “leakiness” (retention of the ability to form AFs) of some of the mutations could result from the presence of functional homologs in the cell or, in some cases, homologs in the AF cluster as will be discussed below. The genes that give a “leaky” phenotype upon disruption are listed in Table 1.

**Table 1:** Comparison of genes in the *A. flavus* aflatoxin gene clusters. The leaky mutation phenotype is defined as a knockout transformant that still produces some aflatoxin. Derailment products are by-product metabolites that in some cases cannot be converted directly to aflatoxins by feeding experiments [16,24,33].

Non-leaky mutations	Leaky mutations	Stable metabolites	Derailment products
*aflR*	*hypB*	Norsolorinic acid (NA)	Averufanin
*aflJ*	*hypC*	Averantin (AVN)	Averythrin
*avnA*	*hypD*	Hydroxyaverantin (HAVN)	Nidurufin
*avfA?*	*hypE*	Oxoaverantin (OAVN)	6-Deoxyversicolorin A
*estA*	*nor-1*	Averufin (AVF)	Versicolorone
*pksA*	*norA*	Hydroxyversicolorone (HVN)	Versicolorol
*hexA/hexB*	*norB*	Versicolorin hemiacetal acetate (VHA)	Versiconol
*cypA*	*ordB*	Versiconal hemiacetal (VAL)	Versiconal
*cypX*	*nadA*	Versicolorin B (VERB)	versiconol acetate
*moxY*	*vbs*	Versicolorin A (VERA)	Sterigmatin
*ver-1*		Demethylsterigmatocystin (DMST)	Aflatoxicol
*verA*		DihydroDMST (DHDMST)	Deoxyaflatoxin
*verB*		Sterigmatocystin (ST)	
*omtA*		DihydroST (DHDMST)	
*omtB*		O-methylsterigmatocystin (OMST)	
*ordA*		DihydroOMST (DHOMST)	
*aflY*		11-HydroxyOMST (HOMST)	
		11-HydroxyDHOMST (DHHOMST)	
		Aflatoxin B_1_, B_2_	
		Aflatoxin G_1_, G_2_	

Although the structures of most of the AF precursors have been known for many years [34], and have helped define the biosynthetic steps, where the presumed precursor is sufficiently unstable to be isolated, identification of which enzyme catalyzes which step may be unclear. Furthermore, possible precursors might not survive the isolation process or the acidic growth medium, with pH values between 3 and 6, in which AF production occurs in most of the known producing organisms. Besides “leakiness” some of the mutations give derailment products that can be mistaken for precursors. Both known stable metabolites and characterized derailment products are also listed in Table 1. In AF biosynthesis 18 true precursor metabolites have been identified. In several of the AF biosynthesis steps, knockout of different genes led to accumulation of the same precursor metabolite, thereby obscuring the relationships among the enzymes implicated in the conversion process for that step.

As mentioned above, some of the conversion steps require multiple precursors and, based on structure, some of these are unlikely to be sufficiently stable to be isolable. By knowing, or guessing at, probable enzyme functionalities based on sequence comparisons, we have attempted to assign roles for predicted enzymes encoded by genes in the aflatoxin cluster whose functions have not yet been assigned. Wherever possible, experimental data is given to provide added evidence for the assignments. 

## 3. Formation of Norsolorinic Acid (NA), the First Stable Metabolite in AF Biosynthesis

**Scheme 1:**
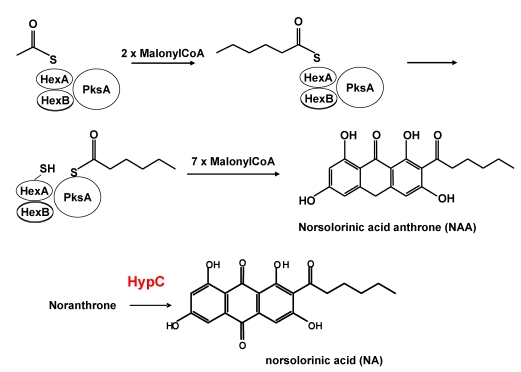
Formation of norsolorinic acid anthrone as the first product of polyketide biosynthesis.

The predicted product from polyketide biosynthesis is norsolorinic acid anthrone (NAA) (Scheme 1). It had been widely assumed that NAA is converted to NA spontaneously, by non-enzymatic O_2_ oxidation [22,35,36,37,38,39]. Oxidation of bacterial anthrones is known to be catalyzed by an unusual low molecular weight (less that 20 kDa) monooxygenase that does not require a co-factor [40,41]. The catalytic active site of this protein consists of amino acids, NXXQWESQAY, near the middle of the protein about 60-70 amino acids from the amino terminus. The single Trp residue in this catalytic motif is assumed to activate the anthrone and the oxidation by molecular oxygen is then mediated by a neighboring Arg residue about 14 aa closer to the carboxyl end of the enzyme. The Trp residue involved in the catalysis may form a charge transfer complex with the anthrone enabling a more favorable energetic state for oxygen addition. A presumed anthrone oxidase involved in conversion of emodin anthrone to emodin was partially purified from a microsomal fraction from *Aspergillus terreus* [42,43]. Its reported size of 50 kDa is much larger than that of the bacterial proteins (14 kDa). Its amino acid and the gene coding sequence have not yet been reported. 

**Figure 2:**
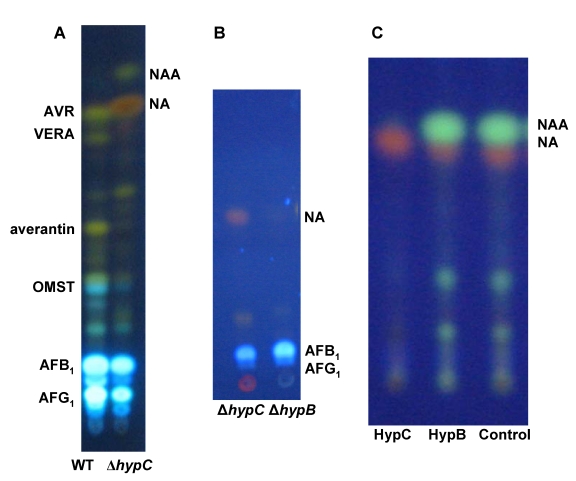
Evidence that HypC is the norsolorinic acid anthrone (NAA) oxidase. (A) Comparison by TLC of extracts of wild type *A. parasiticus* BN009E and ∆*hypC* transformant cultures. (B) extracts of 48 hr *A. parasiticus* ∆*hypC* and ∆*hypB* cultures, and (C) metabolites produced by incubation of synthetic NAA with purified HypC and HypB expressed in *E. coli*. (NA = norsolorinic acid).

In AF clusters from section *Flavi* fungi (*A. parasiticus*, *A. flavus*, *A. nomius* and others) two hypothetical genes, *hypC* and *hypB*, are predicted to encode closely related 17 kDa proteins. In *A. nidulans* and *A. ochraceoroseus* only one copy of these hypothetical genes was found in the cluster. Based on EST data, the genes *hypC* and *hypB* (originally named *hypB1* and *hypB2*), are expressed under conditions conducive to AF production, but not under non-conducive conditions. The putative promoter regions for both genes have TCGN_5_CGA motifs, the canonical binding sequence for the AF pathway transcriptional regulatory factor, AflR. Disruption of *hypC* gave mutant fungi that accumulated excess amounts of NA, a small amount of NAA, and lower amounts of AFs than did selection marker-transformed recipient fungi (Figure 2A) whereas no precursor metabolite accumulated after disruption of *hypB* (Figure 2B). Expression of *hypC* (codon-optimized for *E. coli* expression) in bacteria gave a protein fraction that catalyzed conversion of synthetic NAA to NA (Figure 2C). Incubation of NAA with HypB, also expressed in *E. coli*, did not catalyze this conversion. These results demonstrate that HypC, but not HypB, is the anthrone oxidase involved in the catalytic conversion of NAA to NA. The ability of the disruption mutant to still produce AFs suggests that either a *hypC* homolog is able to complement the defect or non-enzymatic oxidation is a significant alternative pathway allowing the enzymatic loss to be bypassed. 

**Figure 3:**
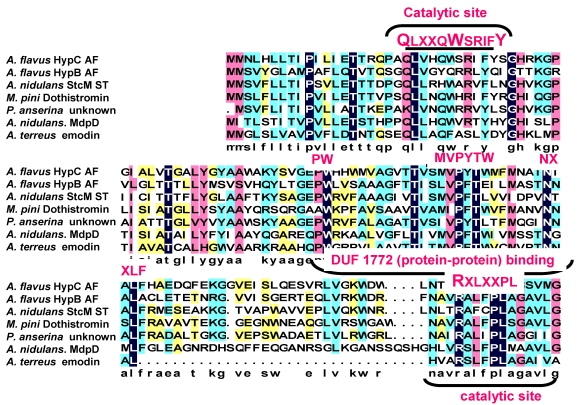
Alignment of HypC orthologs from fungi known to produce AF precursors or related metabolites. Putative catalytic sites are shown above and below the alignment in red text.

A tBlastN search of the Broad Institute Aspergillus comparative database (http://www.broad.mit.edu/annotation/genome/aspergillus_terreus/MultiHome.html) or the Multi-fungi database (http://www.broadinstitute.org/cgi-bin/annotation/fgi/blast_page.cgi), containing sequenced genomes of many fungal species, revealed numerous HypC orthologs. Only some of potential orthologs are listed in the alignment in Figure 3. Included in the list is StcM, the candidate anthrone oxidase from *A. nidulans*, a species that produces the AF precursor, ST. In fact, all of the known producers of AF precursor-like metabolites were found to have genes predicted to encode proteins similar to the small HypC-like anthrone oxidase. Comparison of the predicted amino acid sequence of suspected HypC orthologs revealed potential catalytic sites similar to those characterized in known bacterial anthrone oxidases. In the comparison of fungal anthrone oxidases, of the several possible Trp residues, the one that fits best with the bacterial active site has the motif QLXXQWSRIFY. Since Gln (Q) and Asp (N) have interchangeable properties, the assignment of this motif as the catalytic site is plausible. HypB, while also possessing several key Trp residues is missing this one, thus providing an explanation for why HypB is unable to catalyze NAA oxidation in our *in vitro* studies.

The anthrone oxidase from *A. terreus* was characterized previously as a much larger molecule [42]. We suggest that the HypC and HypC-like proteins are the actual fungal anthrone oxidases and have a size similar to those of bacteria. During the studies of the oxidation of synthetic NAA and emodin anthrone (also produced synthetically, results not shown) we found that the substrate was quite sensitive to buffer conditions and to air oxidation when the pH of the environment was sufficiently basic (pH 7.5 or higher). Under these conditions the substrate was converted, partially to a red pigment that remained at the origin under the conditions used for TLC. Previously, the anthrone oxidase assays were done by spectophotometric determination of the red shift that occurs when emodin anthrone is converted to emodin [42]. Formation of the red pigment at the origin observed by TLC would also give a red shift that could have been easily mistaken for catalytic conversion. Furthermore, a smear of protein on the SDS-PAGE gel shown in their study suggests that their sample was quite impure and, therefore, molecular weight was probably not accurately determined in their electrophoretic analysis. Since no other characterization was reported, we strongly suggest that HypC is the actual fungal anthrone oxidase and closely resembles its bacterial counterparts in size and in activity.

## 4. Assignment of a Role for AvfA in Averufin Oxidation

The chemical steps in the conversion of averufin to hydroxyversicolorone have been partially worked out [44,45,46,47] and recently, the cytochrome P450 oxidoreductase CypX (AflV), has been unequivocally assigned to this conversion [46]. Based on complementation studies, two groups found that the gene *avfA (aflI)* is also involved in this conversion [23,48]. AvfA encodes a presumed flavin dependent oxidoreductase of uncertain catalytic functionality. Both knockout of *cypX* or *avfA* led to accumulation of averufin (AVF), with loss of accumulation of metabolites beyond AVF in the biosynthesis. A precise role for AvfA in the oxidation of AVF has never been determined.

**Scheme 2:**
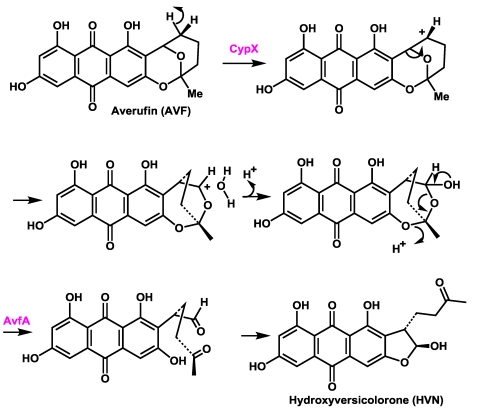
Steps in the conversion of averufin to hydroxyversicolorone.

There are stereochemical constraints on the rearrangement. As discussed by Townsend, *et al.* [49], the rearrangement involves a 1,2-aryl shift. The reaction is probably initiated by hydride abstraction from the 2’-carbon by the cytochrome P450, CypX, which may be facilitated by interaction with the aryl hydroxyl at carbon-1 (Scheme 2). Oxidation at one of the secondary carbons of AVF is consistent with the expected functionality of CypX, which has closest similarity to a cytochrome P450 monooxygenase involved in oxidation of a secondary carbon of a trichothecene precursor. This allows rearrangement to proceed across one face of the closed form of the ketal side chain of AVF to be consistent with the stereochemistry of the resulting product, hydroxyversicolorone (HVN), even though there is a required ring-opening reaction, in which the stereochemistry of the rearrangement could be lost. Townsend, *et al*. convincingly showed that a Favorskii-type rearrangement via formation of a cyclopropanone ring is highly unlikely [45]. Furthermore they proved that nidurufin, a known hydroxylation product of AVF, is not a precursor but rather a shunt metabolite [50]. Nidurufin does not accumulate in cultures from gene knockouts of *cypX* or *avfA*, confirming their results [46]. The bridged intermediate in Scheme 2 would be expected to open to give the stereochemically correct configuration of the aldehyde. Since the aldehyde has not been identified as an intermediate in the conversion, it is possible that oxidation of the hydrated CypX intermediate may be catalyzed by AvfA as shown in Scheme 2. To explain why only AVF accumulates in transformants lacking AvfA, we hypothesize that AvfA may form a complex with CypX that aids the electron transfer to flavin necessary for formation of the carbonium ion. 

**Scheme 3:**
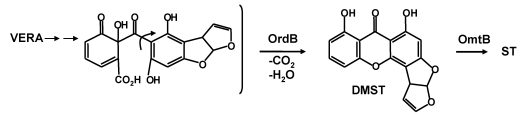
The oxidative decarboxylation and dehydration catalyzed by the AvfA homolog OrdB (AflX) leading to xanthone ring formation in DMST.

Catalysis of the ring closure to form the hydroxyfuran is consistent with the expected functionality for AvfA. Search of the fungal databases for proteins similar to AvfA identified OrdB (AflX) as a high-scoring match. OrdB possesses a catalytic motif identified as a NADP:flavin oxidoreductase. We have provided evidence that OrdB functions in the conversion of versicolorin A (VERA) to DemethylST (DMST) by catalyzing the oxidative decarboxylation and ring-closure of the Baeyer-Villiger intermediate that results from HypA (AflY)-catalyzed oxidation [51,52] (Scheme 3). The HypA intermediate has not been isolated and gene disruptions of either OrdB or HypA led to accumulation of VERA. Although the NADP:flavin catalytic domain is not identified in AvfA, similarity to OrdA suggests retention of at least partial function. It is plausible therefore that, since oxidative decarboxylation is not required for ring closure of the intermediate open-ring hydroxyaldehyde to give the hydroxyfuran, AvfA catalyzes only alcohol oxidation of the HVN precursor by retaining the dehydration functionality of OrdB. It is highly unlikely that CypX alone carries out the oxidation, hydration, and alcohol dehydrogenase closure steps.

## 5. The Last Steps in AF Formation

**Scheme 4:**
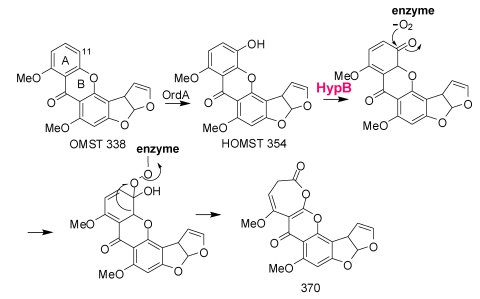
Involvement of HypB in the second oxidation step leading from OMST to the 370 Da 7-membered ring lactone precursor for AFB1 and AFG1 formation.

Formation of AFB_1_ is initiated by the cytochrome P450 monooxygenase, OrdA (AflQ), mediated oxidation of *O*-methylsterigmatocystin (OMST) (Scheme 4) [53,54]. Knockout of the gene for this enzyme led to accumulation of OMST. Feeding of OMST to yeast cells containing *ordA* allowed formation of AFB_1_ [53,55], a result suggesting that OrdA is the only enzyme required for this complicated multistep chemical conversion. Townsend has shown that 11-hydroxyOMST (HOMST) is also an AFB_1_ precursor and is the likely initial product of OMST oxidation by OrdA. Further oxidative rearrangement to the coumarin ring system in AFB_1_ must be consistent with the following observations: 1) NADPH is utilized in the conversion; 2) an “NIH hydride shift” must occur to allow the C-11 hydrogen to be retained; 3) an oxygen atom and C-11 in the A-ring of OMST are lost as carbon dioxide; and 4) an oxygen atom incorporated into the pyrone ring (Scheme 4) is retained [55]. The “reuse” of OrdA for the second required oxidation step is implausible. Not only would the enzyme have very different substrate specificity from that of the first oxidation, but the ring opening that follows would not be a catalyzed step. In the conversion originally postulated [55], after oxidation of HOMST a 370 Da seven-member ring lactone is formed (Scheme 4). The 370 Da intermediate accumulates in detectable amounts in cultures of *A. parasiticus* with disrupted *norA*, *nadA*, or *norB* [33]. HOMST another postulated intermediate in the conversion was also detected in extracts of these knockout cultures [33]. 

We have reinterpreted a scheme offered by Udwary, *et al.* (Scheme 9 of reference [55]) to include involvement of oxidative enzymes encoded by three of the uncharacterized genes in the AF biosynthesis gene cluster. In Scheme 4, rather than the ring-opening of HOMST involving a second OrdA-catalyzed oxidation, oxidation by HypB, the HypC homolog, could introduce an oxygen into the keto-tautomer of HOMST, followed by rearrangement to the 370 Da intermediate. Such an oxidation would be similar to the anthrone oxidation by HypC, namely, oxidation by molecular oxygen at an activated phenolic ring. As Udwary, *et al*. suggested [55], the oxidation route shown in their Scheme 9, involving nucleophilic attack by molecular oxygen on the keto tautomer of HOMST would require little motion of the substrate relative to the catalytic center and would lead directly to Baeyer-Villiger-like rearrangement to the seven-membered ring 370 Da lactone. Since ST is not a substrate for OrdA it may be that the presence of the methoxy group in OMST enforces the hydroquinone oxidation state in the A-ring and maintains the syn-geometry after ring-opening to favor five-member ring formation in the proposed decarboxylative aldol reaction. The 370 Da intermediate must be sufficiently stable to be identifiable by mass spectrometry and to be the probable substrate for CypA-catalyzed oxidation required for formation of AFG_1_ [56]. 

**Scheme 5:**
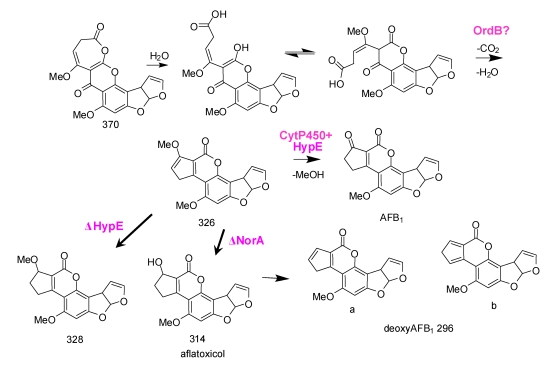
The last steps in AFB_1_ formation: HypE and NorA oxidation of putative intermediates.

Further rearrangements that allow formation of AFB_1_ are shown in Scheme 5. Hydrolysis and ring-opening of the 370 Da lactone could occur spontaneously or involve the action of hydrolytic enzymes encoded by genes that are not part of the AF cluster. In the absence of isolable intermediates from gene disruption studies, assignments of catalytic steps in Scheme 5 is based mainly on fitting plausible reaction steps in the conversion to the enzymes’ putative functions. Just as we recently reported that NadA and NorB (AflF) are involved in AFG_1_ formation from a CypA (AflU)-created intermediate [56], we now suggest that HypE and the homolog to NorB, NorA (AflE), are involved in the last steps in AFB_1_ formation. OrdB, described above, may catalyze an oxidative decarboxylation/dehydration to give the 326 Da metabolite shown in Scheme 5.

**Figure 4:**
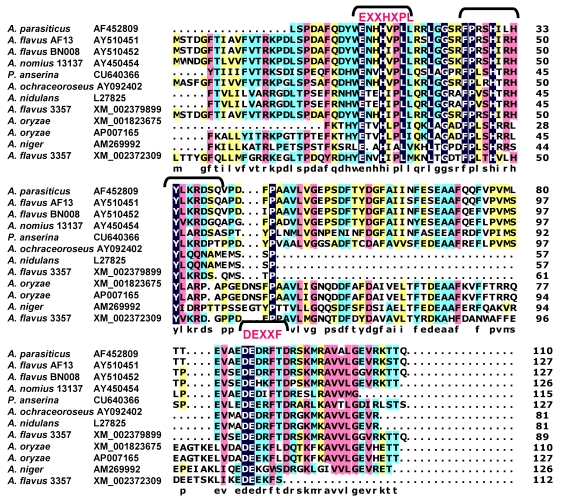
Sequence alignment of HypE orthologs from fungi. Conserved amino acids in the EthD domain are bracketed.

Demethylation of the 326 Da metabolite to AFB_1_ is also likely to be enzymatically catalyzed. To produce either AFB_1_ or AFG_1_ the A-ring methyl residue derived from OMST must be removed. Whether or not demethylation occurs concomitantly with decarboxylation and ring-closure is not known. However, the enzyme, HypE, which is part of the AF cluster, has a catalytic domain that would be suitable for ether hydrolysis. Blast search of the translated nucleotide database revealed the presence of orthologs of HypE in many fungi (Figure 4). These proteins possess an ethD domain, a domain that previously was only reported in a protein from bacteria [57] that is required for ethyl-t-butyl ether degradation [58]. The specific role of the 103 amino acid bacterial protein in ethyl-t-butyl ether degradation has not been determined, but it is known that it works in conjunction with a bacterial cytochrome P450 oxidase. 

Holmes found that disruption of *hypE* in *A. flavus* gave isolates that accumulated a compound with the intense blue fluorescence characteristic of aflatoxins, but which migrated much faster than AFB_1_ or AFG_1_ on TLC (Robert Holmes’ PhD Thesis: http://www.lib.ncsu.edu/theses/available/etd-08182008-112539/unrestricted/etd.pdf [57]). A preliminary analysis of the metabolite mixture from a *hypE* knockout culture by mass spectrometry identified a compound with mass 328. A plausible candidate for this compound is the 328 Da methyl ether that could be a reduction product of the 326 Da enol ether expected to result from ring closure following decarboxylation as shown in Scheme 5. Upon oxidation of the methyl residue with an unknown cytochrome P450 enzyme and the ethD domain protein (HypE) the resulting oxidized product from the 326 Da intermediate would lose the methyl as formaldehyde to directly give AFB_1_. The 328 Da compound, after demethylation, would require an additional oxidation step to give AFB_1_. A similar series of steps can be envisioned for formation of AFG_1_ upon reduction, ether hydrolysis, and re-oxidation. An AF cluster-encoded aryl alcohol dehydrogenase, NorA, may catalyze the re-oxidation as described below. HypE may act in conjunction with one of the five AF biosynthesis cluster cytochrome P450 monooxygenases (possibly CypX or OrdA) to hydroxylate the methyl ether to give, first, an acetal intermediate and then, AFB_1_, after loss of formaldehyde.

Previously, the role of NorA in AF biosynthesis was not defined [59]. Orthologs of genes encoding NorA and NorB are found in non-aflatoxigenic fungi, including yeast, so it is possible that such aryl alcohol dehydrogenases have several functions in biosynthesis of secondary or primary metabolites. Mutants of *norA* in *A. parasiticus* produced the same mixture of metabolites as the wild-type fungi while disruptants of *norA* in *A. flavus* accumulated a bright blue fluorescent metabolite in addition to smaller quantities of AFB_1_ compared to the untransformed control. The bright blue fluorescent metabolite was identified as deoxyAFB_1_ by its mass spectrum (m/z = 297) and its co-migration on TLC and HPLC with deoxyAF prepared by dehydration of aflatoxicol (AFOH) [60]. To account for the formation of deoxyAF, in the absence of NorA, AFOH is formed in *norA* disruptant cultures, either prior to or after formation of AFB_1_, and is dehydrated in the acidic growth medium (Scheme 5). In these studies no evidence was found for accumulation of the 326 Da metabolite, but such a metabolite was obtained by knockout of *norB* in *A. parasiticus* [33]. Both the predicted 328 and 326 Da ethers would be expected to be rapidly demethylated to yield AFB_1_ or AFOH, respectively, by HypE-mediated cytochrome P450 monooxygenase oxidation. NorA may catalyze the oxidization of AFOH back to AFB_1_ and, thereby serve as a maintenance oxidase. 

## 6. Possible Involvement of HypD in AF Accumulation and Fungal Development

**Figure 5:**
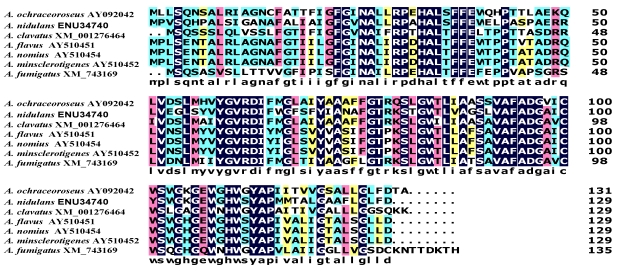
Alignment of HypD proteins from different fungi.

**Figure 6:**
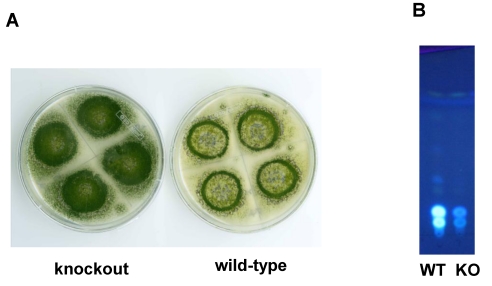
Characteristics of *A. parasiticus* BN009E Δ*hypD* cultures compared to untransformed cultures (A) colony morphology (B) metabolite profile on TLC.

Based on expressed sequence tag data, another small gene, *hypD*, predicted to encode a 129 Da integral membrane binding protein with a DUF6 domain, was discovered in the AF cluster. The high degree of sequence conservation of HypD orthologs in many fungi (Figure 5) suggests these proteins have an important functional role in fungi. Disruption of *hypD* in AF-producing *A. parasiticus* gave isolates that had markedly increased ability to sporulate compared to the wild-type control (Figure 6) but diminished yield of both AFB_1_ and AFG_1_. These results suggest that HypD affects processes that involve both development and secondary metabolism. Integral membrane proteins can act as permeases or metabolite transporters or function as subunits of proteins such as oxidoreductases or glucan synthases [61,62,63]. At this point we cannot say if HypD plays a role in AF efflux or cytochrome P450 enzyme activity or both. Consistent with the function of integral membrane proteins HypD might assist OrdA in oxidation of OMST [64,65,66] and enable AF efflux from the cell. It is known that AF is mostly excreted from fungal cells, but the only other AF cluster gene with homology to a transporter, namely *aflT*, is not involved in AF efflux [67]. It is possible that HypD functions as a permease and that in knockout cultures, in the absence of HypD function, causes feedback inhibition of AF biosynthesis with a concomitant increase in formation of conidia. There is known inverse relationship between the developmental and secondary metabolite production pathways that would explain the reduction in AF formation and the increase in conidia in *hypD* knockout cultures [68,69].

**Figure 7:**
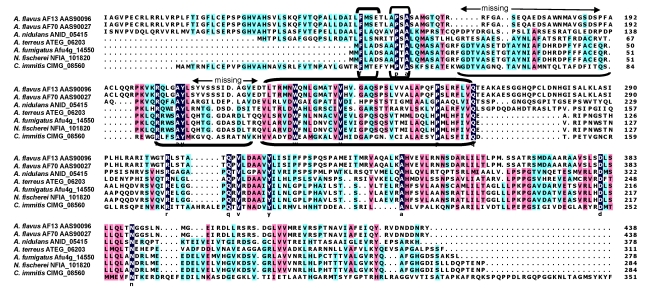
Alignment of AflJ orthologs from different fungi showing amino acids involved in the methyltransferase-2 domain (bracketed at bottom) and missing in the domains of AflJ orthologs from ST and AF-producing species.

## 7. New Insights into the Involvement of AflJ in AF Biosynthesis Regulation

Previous research sought a role for AflJ (AflS) in regulation of transcription of AF biosynthesis genes [70,71,72,73]. It was established in 1998 that disruption of *aflJ* caused loss of AF production as well as the production of all AF biosynthesis proteins. There was some confusion as to whether or not transcripts were still made from AF genes in *aflJ* disruptants, but attempts to complement *aflJ* mutants by feeding AF precursors were unsuccessful. *aflJ* and *aflR* share a common intergenic region and *aflJ* transcription is regulated by AreA, the global transcription factor for nitrate utilization [74], as well as by the pathway-specific transcription regulatory protein, AflR. These results suggested that AflJ plays a regulatory role in biosynthesis. By yeast two-hybrid studies, Chang found that AflJ bound to AflR [70]. We expanded these studies and found that AflJ also interacts with another protein that acts globally as a regulator of secondary metabolite biosynthesis, namely the methyltransferase, LaeA [25,75,76,77,78,79]. Transformant *A. parasiticus* isolates possessing additional copies of AflJ produced higher levels of AF and precursor metabolites [70]. AflJ has three membrane spanning domains and a microbodies signaling sequence at its C-terminal end. By tBlastN analysis, AflJ and AflJ-like proteins were found only in fungi. No conserved domains were detected. However, some of the AflJ-orthologs identified by the tBlastN search contain a conserved methyltransferase-2 (*O*-methyltransferase) domain. When these and the AflJ orthologs from AF-producing fungi were aligned, several regions in AflJ that are conserved in methyltransferase-2 domain proteins were missing (Figure 7). This explains why Blast search was unable to pick up the methyltransferase-2 domain in AflJ. Nonetheless, a partial domain could still retain biological function, namely, it could bind to another methyltransferase, *e.g.*, LaeA, and become a key nuclear protein for activating specific secondary metabolism gene clusters [25,75,76,77,78,79]. While unproven, LaeA may modulate chromatin activity at the site of secondary metabolism gene clusters. We suggest that LaeA requires a specific interacting partner, namely AflJ or an AflJ-like protein to allow it to target specific secondary metabolite gene clusters. AflJ is a good candidate for such a partner. It is expected to be nuclear membrane bound, interacts with AflR, and aids formation of the transcription complex. All that is still needed is to ensure a region of open chromatin to allow robust transcriptional activity. Loss of high level transcriptional activity of AF cluster genes has been observed when the genes are cloned into a different locus. When non-AF biosynthesis genes are cloned into the AF cluster they have high levels of transcription [80,81]. Another role for AflJ could be related to its ability to bind to components of the de-ubiquitination pathway, for example CsnF and Nedd8, components of the COP9 signalosome [82,83], a multiprotein complex that prevents protein degradation due to ubiquitination [84,85,86]. Both activation of transcription and stabilization of the protein products formed from the mRNA are necessary to assure the production of high levels of secondary metabolites.

## 8. Conclusions

The biosynthetic steps leading to formation of AFB_1_ and G_1_ from OMST most likely involve multiple enzymes rather than just OrdA for catalysis as was previously suggested. Until the catalytic properties of the individual enzymes are characterized in detail, their role in the conversion schemes reported in this review must remain speculative. To date few such detailed characterizations have been done. Our data allow functional classification of certain previously hypothetical enzymes. HypC and HypC-like proteins are probable oxygenases that are able to catalyze the introduction of oxygen into activated aryl moieties. AflJ and AflJ-like proteins are associated with many secondary metabolite biosynthesis gene clusters in fungi and may form complexes with proteins that affect chromatin activity, such as LaeA. NADH:flavin reductases similar to OrdB and AvfA are often associated with secondary metabolite clusters and our data suggests they are involved in ring closure steps with or without decarboxylation or loss of water. These assignments of function should help to better understand the roles of genes commonly associated with secondary metabolite gene clusters that are not now well understood.

## References

[1] Bhatnagar D., Cary J.W., Ehrlich K., Yu J., Cleveland T.E. (2006). Understanding the genetics of regulation of aflatoxin production and *Aspergillus flavus* development. Mycopathologia.

[2] Chang P.K., Matsushima K., Takahashi T., Yu J., Abe K., Bhatnagar D., Yuan G.F., Koyama Y., Cleveland T.E. (2007). Understanding nonaflatoxigenicity of *Aspergillus sojae*: A windfall of aflatoxin biosynthesis research. Appl. Microbiol. Biotechnol..

[3] Georgianna D.R., Payne G.A. (2009). Genetic regulation of aflatoxin biosynthesis: From gene to genome. Fungal Genet. Biol..

[4] Hedayati M.T., Pasqualotto A.C., Warn P.A., Bowyer P., Denning D.W. (2007). *Aspergillus flavus*: Human pathogen, allergen and mycotoxin producer. Microbiology.

[5] Holmes R.A., Boston R.S., Payne G.A. (2008). Diverse inhibitors of aflatoxin biosynthesis. Appl. Microbiol. Biotechnol..

[6] Horn B.W. (2007). Biodiversity of *Aspergillus* section *Flavi* in the United States: A review. Food Addit. Contam..

[7] Keller N.P., Turner G., Bennett J.W. (2005). Fungal secondary metabolism-from biochemistry to genomics. Nat. Rev. Microbiol..

[8] Richard J.L. (2007). Some major mycotoxins and their mycotoxicoses-an overview. Int. J. Food Microbiol..

[9] Yin Y.N., Yan L.Y., Jiang J.H., Ma Z.H. (2008). Biological control of aflatoxin contamination of crops. J. Zhejiang Univ. Sci. B.

[10] Yu J., Cleveland T.E., Nierman W.C., Bennett J.W. (2005). *Aspergillus flavus* genomics: Gateway to human and animal health, food safety, and crop resistance to diseases. Rev. Iberoam. Micol..

[11] Geiser D.M., Dorner J.W., Horn B.W., Taylor J.W. (2000). The phylogenetics of mycotoxin and sclerotium production in *Aspergillus flavus* and *Aspergillus oryzae*. Fungal Genet. Biol..

[12] Cotty P.J., Mellon J.E. (2006). Ecology of aflatoxin producing fungi and biocontrol of aflatoxin contamination. Mycotoxin Res..

[13] Wicklow D.T., Wilson D.M., Nelsen T.C. (1993). Survival of *Aspergillus flavus* sclerotia and conidia buried in soil in Illinois and Georgia. Phytopathology.

[14] Gourama H., Bullerman L.B. (1995). *Aspergillus flavus* and *Aspergillus parasiticus*: Aflatoxigenic fungi of concern in foods and feeds: A review. J. Food Prot..

[15] Robens J. (2001). The costs of mycotoxin management to the USA: Management of aflatoxins in the United States. APSnet Feature.

[16] Yabe K., Nakajima H. (2004). Enzyme reactions and genes in aflatoxin biosynthesis. Appl. Microbiol. Biotechnol..

[17] Ehrlich K.C., Yu J., Cotty P.J. (2005). Aflatoxin biosynthesis gene clusters and flanking regions. J. Appl. Microbiol..

[18] Yu J., Cleveland T.E., Rimando A.M., Baerson S.R. (2007). *Aspergillus flavus* genomics for discovering genes involved in aflatoxin biosynthesis. Polyketides: Biosynthesis, Biological activity, and Genetic Engineering.

[19] Bhatnagar D., Ehrlich K.C., Cleveland T.E., Bhatnagar D., Lillehoj E.B., Arora D.K. (1992). Oxidation-reduction reactions in biosynthesis of secondary metabolites. Mycotoxins in Ecological Systems.

[20] Cary J.W., Ehrlich K.C. (2006). Aflatoxigenicity in *Aspergillus*: Molecular genetics, phylogenetic relationships and evolutionary implications. Mycopathologia.

[21] Dutton M.F. (1988). Enzymes and aflatoxin biosynthesis. Microbiol. Rev..

[22] Minto R.E., Townsend C.A. (1997). Enzymology and molecular biology of aflatoxin biosynthesis. Chem. Rev..

[23] Yu F.L. (1977). Mechanism of aflatoxin B1 inhibition of rat hepatic nuclear RNA synthesis. J. Biol. Chem..

[24] Yu J., Chang P.K., Ehrlich K.C., Cary J.W., Bhatnagar D., Cleveland T.E., Payne G.A., Linz J.E., Woloshuk C.P., Bennett J.W. (2004). Clustered pathway genes in aflatoxin biosynthesis. Appl. Environ. Microbiol..

[25] Bok J.W., Balajee S.A., Marr K.A., Andes D., Nielsen K.F., Frisvad J.C., Keller N.P. (2005). LaeA, a regulator of morphogenetic fungal virulence factors. Eukaryot. Cell.

[26] Bok J.W., Hoffmeister D., Maggio-Hall L.A., Murillo R., Glasner J.D., Keller N.P. (2006). Genomic mining for *Aspergillus* natural products. Chem. Biol..

[27] Bradshaw R.E., Zhang S. (2006). Biosynthesis of dothistromin. Mycopathologia.

[28] Brobst S.W., Townsend C.A. (1994). The potential role of fatty-acid initiation in the biosynthesis of the fungal aromatic polyketide aflatoxin b-1. Can. J. Chem..

[29] Brown D.W., Yu J.H., Kelkar H.S., Fernandes M., Nesbitt T.C., Keller N.P., Adams T.H., Leonard T.J. (1996). Twenty-five coregulated transcripts define a sterigmatocystin gene cluster in *Aspergillus nidulans*. Proc. Natl. Acad. Sci. USA.

[30] Carbone I., Ramirez-Prado J.H., Jakobek J.L., Horn B.W. (2007). Gene duplication, modularity and adaptation in the evolution of the aflatoxin gene cluster. BMC Evol. Biol..

[31] Cary J.W., Ehrlich K.C., Beltz S.B., Harris-Coward P., Klich M.A. (2009). Characterization of the *Aspergillus ochraceoroseus* aflatoxin/sterigmatocystin biosynthetic gene cluster. Mycologia.

[32] Jenke-Kodama H., Sandmann A., Muller R., Dittmann E. (2005). Evolutionary implications of bacterial polyketide synthases. Mol. Biol. Evol..

[33] Ehrlich K.C., Scharfenstein L.L., Montalbano B.G., Chang P.-K. (2008). Are the genes *nadA* and *norB* involved in formation of aflatoxin G_1_. Int. J. Mol. Sci..

[34] Maggon K.K., Gupta S.K., Venkitasubramanian T.A. (1977). Biosynthesis of aflatoxins. Bacteriol. Rev..

[35] Bennett J.W., Chang P.K., Bhatnagar D. (1997). One gene to whole pathway: The role of norsolorinic acid in aflatoxin research. Adv. Appl. Microbiol..

[36] Trail F., Chang P.K., Cary J., Linz J.E. (1994). Structural and functional analysis of the *nor-1* gene involved in the biosynthesis of aflatoxins by *Aspergillus parasiticus*. Appl. Environ. Microbiol..

[37] Weissman K.J. (2008). Biochemistry. Anatomy of a fungal polyketide synthase. Science.

[38] Wilkinson J.R., Yu J., Abbas H.K., Scheffler B.E., Kim H.S., Nierman W.C., Bhatnagar D., Cleveland T.E. (2007). Aflatoxin formation and gene expression in response to carbon source media shift in *Aspergillus parasiticus*. Food Addit. Contam..

[39] Yabe K., Yan P.S., Song Y., Ichinomiya M., Nakagawa H., Shima Y., Ando Y., Sakuno E., Nakajima H. (2008). Isolation of microorganisms and substances inhibitory to aflatoxin production. Food Addit. Contam. Part A Chem. Anal. Control Expo. Risk Assess..

[40] Chung J.Y., Fujii I., Harada S., Sankawa U., Ebizuka Y. (2002). Expression, purification, and characterization of AknX anthrone oxygenase, which is involved in aklavinone biosynthesis in *Streptomyces galilaeus*. J. Bacteriol..

[41] Sciara G., Kendrew S.G., Miele A.E., Marsh N.G., Federici L., Malatesta F., Schimperna G., Savino C., Vallone B. (2003). The structure of ActVA-Orf6, a novel type of monooxygenase involved in actinorhodin biosynthesis. Embo. J..

[42] Chen Z.-G., Fujii I., Ebizuka Y., Sankawa U. (1995). Purification and characterization of emodinanthrone oxygenase from *Aspergillus terreus*. Phytochemistry.

[43] Fujii I., Chen Z.G., Ebizuka Y., Sankawa U. (1991). Identification of emodinanthrone oxygenase in fungus *Aspergillus terreus*. Biochem. Int..

[44] Sakuno E., Wen Y., Hatabayashi H., Arai H., Aoki C., Yabe K., Nakajima H. (2005). *Aspergillus parasiticus* cyclase catalyzes two dehydration steps in aflatoxin biosynthesis. Appl. Environ. Microbiol..

[45] Townsend C.A., Christensen S.B., Davis S.G. (1988). Synthesis of averufin and its role in aflatoxin-B1 biosynthesis.

[46] Wen Y., Hatabayashi H., Arai H., Kitamoto H.K., Yabe K. (2005). Function of the *cypX* and *moxY* genes in aflatoxin biosynthesis in *Aspergillus parasiticus*. Appl. Environ. Microbiol..

[47] Yabe K., Chihaya N., Hamamatsu S., Sakuno E., Hamasaki T., Nakajima H., Bennett J.W. (2003). Enzymatic conversion of averufin to hydroxyversicolorone and elucidation of a novel metabolic grid involved in aflatoxin biosynthesis. Appl. Environ. Microbiol..

[48] Yu J., Woloshuk C.P., Bhatnagar D., Cleveland T.E. (2000). Cloning and characterization of *avfA* and *omtB* genes involved in aflatoxin biosynthesis in three *Aspergillus* species. Gene.

[49] Townsend C.A., Isomura Y., Davis S.G., Hodge J.A. (1989). Reaction models of the oxidative rearrangement of averufin to 1'-hydroxyversicolorone - the 1st step in dihydrobisfuran formation in aflatoxin biosynthesis. Tetrahedron.

[50] Townsend C.A., Christensen S.B. (1985). Concerning the role of nidurufin in aflatoxin biosynthesis. J. Am. Chem. Soc..

[51] Cary J.W., Ehrlich K., Bland J.M., Montalbano B. (2006). The aflatoxin biosynthesis cluster gene *aflX*, encodes an oxidoreductase involved in conversion of versicolorin A to demethylsterigmatocystin. Appl. Environ. Microbiol..

[52] Ehrlich K.C., Montalbano B., Boue S.M., Bhatnagar D. (2005). An aflatoxin biosynthesis cluster gene encodes a novel oxidase required for conversion of versicolorin a to sterigmatocystin. Appl. Environ. Microbiol..

[53] Prieto R., Woloshuk C.P. (1997). *ord1*, an oxidoreductase gene responsible for conversion of *O*-methylsterigmatocystin to aflatoxin in *Aspergillus flavus*. Appl. Environ. Microbiol..

[54] Yu J., Chang P.-K., Ehrlich K.C., Cary J.W., Montalbano B., Dyer J.M., Bhatnagar D., Cleveland T.E. (1998). Characterization of the critical amino acids of an *Aspergillus parasiticus* cytochrome P-450 monooxygenase encoded by *ordA* that is involved in the biosynthesis of aflatoxins B_1_, G_1_, B_2_, and G_2_. Appl. Environ. Microbiol..

[55] Udwary D.W., Casillas L.K., Townsend C.A. (2002). Synthesis of 11-hydroxy-*O*-methylsterigmatocystin and the role of a cytochrome P-450 in the final step of aflatoxin biosynthesis. J. Am. Chem. Soc..

[56] Ehrlich K.C., Chang P.-K., Yu J., Cotty P.J. (2004). Aflatoxin biosynthesis cluster gene *cypA* is required for G aflatoxin formation. Appl. Environ. Microbiol..

[57] Holmes R.A. (2008). Characterization of an Aflatoxin Biosynthetic Gene and Resistance in Maize Seeds to *Aspergillus flavus*.

[58] Chauvaux S., Chevalier F., Le Dantec C., Fayolle F., Miras I., Kunst F., Beguin P. (2001). Cloning of a genetically unstable cytochrome P-450 gene cluster involved in degradation of the pollutant ethyl tert-butyl ether by *Rhodococcus ruber*. J. Bacteriol..

[59] Cary J.W., Wright M., Bhatnagar D., Lee R., Chu F.S. (1996). Molecular characterization of an *Aspergillus parasiticus* gene, *norA*, located on the aflatoxin biosynthesis gene cluster. Appl. Environ. Microbiol..

[60] Lau H.P., Chu F.S. (1983). Preparation and characterization of acid dehydration products of aflatoxicol. J. Assoc. Off. Anal. Chem..

[61] Chan S.I., Yu S.S. (2008). Controlled oxidation of hydrocarbons by the membrane-bound methane monooxygenase: The case for a tricopper cluster. Acc. Chem. Res..

[62] Pereira M., Felipe M.S., Brigido M.M., Soares C.M., Azevedo M.O. (2000). Molecular cloning and characterization of a glucan synthase gene from the human pathogenic fungus *Paracoccidioides brasiliensis*. Yeast.

[63] Szczesna-Skorupa E., Kemper B. (2008). Influence of protein-protein interactions on the cellular localization of cytochrome P450. Expert Opin. Drug Metab. Toxicol..

[64] Calvo A.M., Wilson R.A., Bok J.W., Keller N.P. (2002). Relationship between secondary metabolism and fungal development. Microbiol. Mol. Biol. Rev..

[65] Reiss J. (1982). Development of *Aspergillus parasiticus* and formation of aflatoxin B_1_ under the influence of conidiogenesis affecting compounds. Arch. Microbiol..

[66] Wieser J., Yu J.H., Adams T.H. (1997). Dominant mutations affecting both sporulation and sterigmatocystin biosynthesis in *Aspergillus nidulans*. Curr. Genet..

[67] Chang P.K., Yu J., Yu J.H. (2004). *aflT*, a MFS transporter-encoding gene located in the aflatoxin gene cluster, does not have a significant role in aflatoxin secretion. Fungal Genet. Biol..

[68] Amaike S., Keller N.P. (2009). Distinct roles for VeA and LaeA in development and pathogenesis of *Aspergillus flavus*. Eukaryot. Cell.

[69] Roze L.V., Calvo A.M., Gunterus A., Beaudry R., Kall M., Linz J.E. (2004). Ethylene modulates development and toxin biosynthesis in *Aspergillus* possibly via an ethylene sensor-mediated signaling pathway. J. Food Prot..

[70] Chang P.K. (2003). The *Aspergillus parasiticus* protein AFLJ interacts with the aflatoxin pathway-specific regulator AFLR. Mol. Genet. Genomics..

[71] Chang P.K. (2004). Lack of interaction between AFLR and AFLJ contributes to nonaflatoxigenicity of *Aspergillus sojae*. J. Biotechnol..

[72] Du W., Obrian G.R., Payne G.A. (2007). Function and regulation of *aflJ* in the accumulation of aflatoxin early pathway intermediate in *Aspergillus flavus*. Food Addit. Contam..

[73] Meyers D.M., Obrian G., Du W.L., Bhatnagar D., Payne G.A. (1998). Characterization of *aflJ*, a gene required for conversion of pathway intermediates to aflatoxin. Appl. Environ. Microbiol..

[74] Ehrlich K.C., Cotty P.J. (2002). Variability in nitrogen regulation of aflatoxin production by *Aspergillus flavus* strains. Appl. Microbiol. Biotechnol..

[75] Keller N., Bok J., Chung D., Perrin R.M., Keats Shwab E. (2006). LaeA, a global regulator of *Aspergillus* toxins. Med. Mycol..

[76] Bok J.W., Noordermeer D., Kale S.P., Keller N.P. (2006). Secondary metabolic gene cluster silencing in *Aspergillus nidulans*. Mol. Microbiol..

[77] Perrin R.M., Fedorova N.D., Bok J.W., Cramer R.A., Wortman J.R., Kim H.S., Nierman W.C., Keller N.P. (2007). Transcriptional regulation of chemical diversity in *Aspergillus fumigatus* by LaeA. PLoS Pathog..

[78] Kale S.P., Milde L., Trapp M.K., Frisvad J.C., Keller N.P., Bok J.W. (2008). Requirement of LaeA for secondary metabolism and sclerotial production in *Aspergillus flavus*. Fungal Genet. Biol..

[79] Bayram O., Krappmann S., Ni M., Bok J.W., Helmstaedt K., Valerius O., Braus-Stromeyer S., Kwon N.J., Keller N.P., Yu J.H., Braus G.H. (2008). VelB/VeA/LaeA complex coordinates light signal with fungal development and secondary metabolism. Science.

[80] Chiou C.H., Miller M., Wilson D.L., Trail F., Linz J.E. (2002). Chromosomal location plays a role in regulation of aflatoxin gene expression in *Aspergillus parasiticus*. Appl. Environ. Microbiol..

[81] Liang S.H., Wu T.S., Lee R., Chu F.S., Linz J.E. (1997). Analysis of mechanisms regulating expression of the *ver-1* gene, involved in aflatoxin biosynthesis. Appl. Environ. Microbiol..

[82] Busch S., Eckert S.E., Krappmann S., Braus G.H. (2003). The COP9 signalosome is an essential regulator of development in the filamentous fungus *Aspergillus nidulans*. Mol. Microbiol..

[83] Lima J.F., Malavazi I., von Zeska Kress Fagundes M.R., Savoldi M., Goldman M.H., Schwier E., Braus G.H., Goldman G.H. (2005). The *csnD/csnE* signalosome genes are involved in the *Aspergillus nidulans* DNA damage response. Genetics.

[84] Chiba T., Tanaka K. (2004). Cullin-based ubiquitin ligase and its control by NEDD8-conjugating system. Curr. Protein Pept. Sci..

[85] He Q., Cheng P., He Q., Liu Y. (2005). The COP9 signalosome regulates the *Neurospora* circadian clock by controlling the stability of the SCFFWD-1 complex. Genes Dev..

[86] Wimuttisuk W., Singer J.D. (2007). The Cullin3 ubiquitin ligase functions as a Nedd8-bound heterodimer. Mol. Biol. Cell.

